# Costs Associated with Malaria in Pregnancy in the Brazilian Amazon, a Low Endemic Area Where *Plasmodium vivax* Predominates

**DOI:** 10.1371/journal.pntd.0004494

**Published:** 2016-03-31

**Authors:** Camila Bôtto-Menezes, Azucena Bardají, Giselane dos Santos Campos, Silke Fernandes, Kara Hanson, Flor Ernestina Martínez-Espinosa, Clara Menéndez, Elisa Sicuri

**Affiliations:** 1 Universidade do Estado do Amazonas (UEA), Programa de Pós-Graduação em Medicina Tropical, Manaus, Amazonas, Brazil; 2 Fundação de Medicina Tropical Dr. Heitor Vieira Dourado (FMT-HVD), Manaus, Amazonas, Brazil; 3 ISGlobal, Barcelona Centre for International Health Research (CRESIB), Hospital Clínic—Universitat de Barcelona, Barcelona, Spain; 4 Núcleo de Estudos e Pesquisas das Cidades na Amazônia Brasileira da Universidade Federal do Amazonas (NEPECAB/UFAM), Manaus, Amazonas, Brazil; 5 Faculdade Metropolitana de Manaus (FAMETRO), Manaus, Amazonas, Brazil; 6 London School of Hygiene and Tropical Medicine, London, United Kingdom; 7 Centro de Pesquisa Leônidas e Maria Deane/Fundação Oswaldo Cruz, Manaus, Amazonas, Brazil; 8 Health Economics Group, Department of Infectious Disease Epidemiology, School of Public Health, Imperial College London, London, United Kingdom; Mahidol University, THAILAND

## Abstract

**Background:**

Information on costs associated with malaria in pregnancy (MiP) in low transmission areas where *Plasmodium vivax* predominates is so far missing. This study estimates health system and patient costs of MiP in the Brazilian Amazon.

**Methods/Principal Findings:**

Between January 2011 and March 2012 patient costs for the treatment of MiP were collected through an exit survey at a tertiary referral hospital and at a primary health care centre in the Manaus metropolitan area, Amazonas state. Pregnant and post-partum women diagnosed with malaria were interviewed after an outpatient consultation or at discharge after admission. Seventy-three interviews were included in the analysis. Ninety-six percent of episodes were due to *P*. *vivax* and 4% to *Plasmodium falciparum*. In 2010, the total median costs from the patient perspective were estimated at US $45.91 and US $216.29 for an outpatient consultation and an admission, respectively. When multiple *P*. *vivax* infections during the same pregnancy were considered, patient costs increased up to US $335.85, representing the costs of an admission plus an outpatient consultation. Provider direct and overhead cost data were obtained from several sources. The provider cost associated with an outpatient case, which includes several consultations at the tertiary hospital was US $103.51 for a *P*. *vivax* malaria episode and US $83.59 for a *P*. *falciparum* malaria episode. The cost of an inpatient day and average admission of 3 days was US $118.51 and US $355.53, respectively. Total provider costs for the diagnosis and treatment of all malaria cases reported in pregnant women in Manaus in 2010 (N = 364) were US $17,038.50, of which 92.4% (US$ 15,741.14) due to *P*. *vivax* infection.

**Conclusion:**

Despite being an area of low risk malaria transmission, MiP is responsible for a significant economic burden in Manaus. Especially when multiple infections are considered, costs associated with *P*. *vivax* are higher than costs associated with *P*. *falciparum*. The information generated may help health policy decisions for the current control and future elimination of malaria in the area.

## Author Summary

Malaria in pregnancy (MiP) is associated with maternal and foetal morbidity and mortality. In addition to the clinical burden, MiP implies a significant economic burden, but the little available evidence on the economics of MiP is limited to *Plasmodium falciparum* malaria and to the sub-Saharan region. While an increasing interest has been recently devoted to the epidemiology and the clinical consequences of *Plasmodium vivax*, the economics of *P*. *vivax* malaria is neglected. *P*. *vivax* is endemic in Latin America, and Brazil is the country with the highest reported burden of MiP of the American continent. Between 2011 and 2012, we conducted a study in the city of Manaus, Brazilian Amazon, with the aim of estimating costs associated with MiP both from the health provider and the patient perspectives. Despite being an area of low transmission risk, we found that the costs of treating MiP are not negligible. In particular, both from the patient and the provider perspectives, costs underwent a remarkable increase when admission was required and when the cost of subsequent *P*. *vivax* malaria episodes during the same pregnancy occurred. The information generated may help health policy decisions for the current control and future elimination of malaria in the area.

## Introduction

Malaria in pregnancy (MiP) is associated with maternal and foetal morbidity and mortality [1]. Among the clinical consequences of MiP are maternal anemia, low birth weight, prematurity, severe disease, and malaria morbidity in infancy [2–5]. In addition to the clinical burden, MiP implies a significant economic burden [6, 7], but the little available evidence on the economics of MiP is limited to *Plasmodium falciparum* malaria and to the sub-Saharan region [8].

In Brazil, malaria risk is mostly restricted to the Amazon region (99%), and *Plasmodium vivax* is the predominant species, responsible for 84% of malaria cases [9]. The Brazilian National Malaria Control Program relies on prompt diagnosis and treatment of cases [10]. Malaria is a notifiable disease and every confirmed case has to be reported through the National Surveillance System (SIVEP) [10]. Information on pregnancy status is collected for any malaria case reported in women of reproductive age. Although these figures may be overestimated for Brazil as MiP cases are underreported in the continent, in 2008 Brazil notified the 75% (4315/5740) of MiP reported cases in the Americas [11]. Of these, 571 were reported in the Manaus metropolitan area, representing over 13% of the MiP cases in Brazil [12].

Despite the increasing interest devoted to *P*. *vivax* in recent years, little is known on its impact on maternal and foetal health [1]. As for the clinical consequences, the economics of *P*. *vivax* malaria has been neglected [8]. In Brazil, published data on the socio-economic aspects of malaria date from the nineties. At that time attention was focused on *P*. *falciparum* malaria, which was responsible for nearly half of the outpatient cases, and associated with high fatality rates. Most studies focused either on value estimates of productive time losses due to malaria among specific group of workers, mainly miners, or on the costs and the cost-effectiveness of specific malaria control measures [13–18]. Recently, attention has been dedicated to the economic evaluation of diagnostic tools for the identification of the most cost-effective strategy for malaria control in the country [19, 20]. So far, no published data exists on the socio-economic aspects of MiP in Brazil.

The Brazilian National Health System is based on the principle of universal access to care. Malaria treatment is provided only through the National Unified Health System (SUS) and it is free to patients, implying great challenges on the public finance of healthcare [21]. Even if patients do not incur any medical costs, other costs are likely to have a relevant impact on household budgets, such as transportation and indirect costs [6].

This study aims to describe the costs of the treatment of MiP in an endemic setting of the Brazilian Amazon both from the health system and the patient perspectives. This information may be of relevance for health policy decision-making on MiP control and it may have implications for malaria elimination in the region.

## Methods

### Study area and population

This study was carried out at the Fundação de Medicina Tropical Doutor Heitor Vieira Dourado (FMT-HVD) and at the João Avelino Pereira Health Post (JAPHP) in the city of Manaus, Western Brazilian Amazon. Manaus is the most populous city of the Brazilian northern region, with about 2 million inhabitants. Gross domestic product (GDP) per capita in Manaus is US$ 13,695, nearly 20% higher than the national average GDP per capita. Despite this good economic indicator, mainly due to the presence of natural resources in the area, Manaus has the highest poverty rates of the Metropolitan Region’s capitals of the North/Northeast of Brazil [22]. The Gini index for the year 2010 was 0.6334, pointing to high level of inequality in income distribution [23]. The Annual Parasitic Index (API) in Manaus was of 8.87/1,000 inhabitants in 2011, being classified as a low risk malaria transmission area (less than 10 cases/1.000 inhabitants) [24]. In 2011, a total of 215 cases of MiP were reported in the Manaus area. Seventy percent (151/215) were reported by the FMT-HVD, and most of them (96%) were caused by *P*. *vivax* infection [12]. The FMT-HVD is the only public tertiary referral hospital for infectious diseases in the Amazonas state. The institution is a 150-bed hospital, including a passive case detection (PCD) system outpatient clinic, a day-hospital care area, and an intensive care unit. The FMT-HVD is also a referral institution for research and training. The JAPHP is a primary health care centre located in an endemic malaria area in the periphery of Manaus city responsible for 22.5% of local notification in 2010. There is no emergency care or inpatient care at the JAPHP.

### Study design and definitions

This was a cost-of-illness study conducted in the context of the PregVax multicentre collaborative project (UE 201588), a health-facility based cohort study aimed to determine the burden and impact of *P*. *vivax* infection in pregnancy, conducted in five endemic countries: Brazil, Guatemala, Colombia, India and Papua New Guinea.

In the current study, an outpatient malaria episode was defined as an episode that does not require hospital admission. The treatment provided for an outpatient episode is called ambulatory care. Every visit to the health facility, whether for ambulatory care or follow up, is called consultation. At the FMT-HVD, an outpatient malaria episode usually requires three consultations; a first one for diagnosis and treatment, and two additional consultations for follow-up. An inpatient malaria episode requires hospital admission for 3 days plus two follow-up consultations as outpatients after discharge.

Two different approaches were taken to estimate patient and provider costs. Patients’ costs were estimated based on a survey conducted among pregnant women who presented at the FMT-HVD or at the JAPHP and receiving a diagnosis of malaria. Direct and indirect costs were estimated through data collected during the survey. Provider costs were estimated following treatment protocol for *P*. *vivax* and *P*. *falciparum* and by accessing administrative data at the FMT-HVD.

### Ethical considerations

This study received ethical approval by the FMT-HVD Ethics in Research on Humans Committee, and by the Ethical Committee of Clinical Research of the Hospital Clinic of Barcelona (Registration no. 2010/6088) in December 2010. We obtained informed written consent from all participants and from the guardians on behalf of the pregnant women less than 18 years old. The PregVax study was approved by the Brazil National Committee of Ethics in Research (CONEP) (Registration no. 063) in 2009.

### Patients’ data

Pregnant and postpartum women attending the FMT-HVD or the JAPHP from January 2011 to March 2012 diagnosed with clinical malaria (due to any *Plasmodium* species) microscopically confirmed, were invited to participate in the study before leaving the health facility after a consultation or at discharge after admission. After written informed consent was given, women were administered with a questionnaire (S1 Appendix) and personal information, time lost because of the illness, and data on costs for the treatment and prevention (use of bed net, skin repellent, insecticide) of MiP, including direct (medical and non-medical) costs incurred at the health facility were collected. At each contact with the health facility, women were asked about eventual previous treatments sought for the symptoms associated with the same malaria episode.

From the patient perspective the cost of an outpatient consultation and of an admission for a malaria episode were estimated. In the case the same woman was interviewed twice the time gap between two consultations or between a consultation and an admission was observed in order to distinguish between contacts on the same episode and on a new one. The second contact with the health facility was considered as part of a new episode when the time between the first and the second interview was more than 7 days. If the time between two interviews was less than 7 days, the second one was not included in the analysis.

Postpartum period was considered until six weeks after the delivery date. The place of residence for each study woman (neighbourhood) was located on digital maps and distance to the health facility was calculated linearly. Average transportation costs per unit of distance (kilometres) were estimated.

### Providers’ data

Direct medical cost data were gathered by interviewing focal staff from the Department of Health of the State of Amazonas at the municipal, state, and federal levels; as well as focal personnel from the Administration and Malaria Departments of the FMT-HVD. Additional data sources, such as the official databases of the Brazilian Ministry of Health and scientific literature, were also consulted (S1 and S2 Tables) [25].

From the provider perspective, costs of an outpatient (including 3 consultations) and of an inpatient malaria episode (including 3 days admission and 2 follow-up consultations) were estimated. Direct provider costs for a malaria episode included the cost of a thick smear test, the drugs for malaria treatment (including treatment of febrile syndrome), the value of time of health staff, and the cost of food in the case of inpatients.

The cost of a thick smear was assumed to be equal to the one published by Oliveira et al. adjusted for inflation according to the Consumer Price Index for 2011 (S1 Table) [19, 26]. In Oliveira et al. the base-case estimate was taken from Macauley that considered the costs of all supplies used in a PCD system [27]. Microscopes and maintenance, training and the value of time of the staff involved in the malaria diagnosis were added to Macauley’s estimates. The cost of microscopes was based on purchase costs by the Brazilian Ministry of Health (Health Surveillance Foundation) in 2006, annualized based on a 5% depreciation rate and a 15-year average lifespan [19]. The cost of the annual maintenance for one microscope was provided by a supplier of the FMT-HVD. The staff costs for malaria and anemia diagnosis were based on the time allocated to perform each activity reported by the staff of the FMT-HVD. Time was estimated in 20 minutes per diagnosis performed by each staff involved, except the laboratory technician (10 minutes). Training costs was based on one annual 160 hour training for reading blood smears for malaria diagnosis and obtained from the Public Health Central laboratory of the Amazonas (LACEN).

Calculation of malaria treatment costs was based on the current recommendations for case management of MiP in Brazil (S2 Table). The standard treatment for *P*. *vivax* malaria in pregnant women consists of oral chloroquine (CQ) (1500 mg, over three days) followed by weekly prophylactic regimen of CQ (300 mg, over 12 weeks). Radical treatment with primaquine is recommended six months after delivery [8]. The recommendation for treatment of *P*. *falciparum* malaria is quinine plus clindamycin in the first trimester and artemether-lumefantrine in the second and third trimesters. Severe *P*. *vivax* malaria cases are treated as for *P*. *falciparum* cases.

In addition to the malaria treatment, a haemogram was assumed to be performed in both outpatient and inpatient cases. It was assumed that at least a full blood count was performed for the malaria cases managed at the FMT-HVD (outpatient and inpatient cases) but not for those managed at the JAPHP. For *P*. *vivax* cases, one extra outpatient consultation was considered for radical cure six months after delivery. It was considered that malaria treatment during admission was done intravenously, and completed with antimalarials orally after discharge. Antipyretic and antiemetic drugs were considered for both outpatient and inpatient treatments, regardless of their actual use. The patients are advised to use these medications only in the presence of symptoms, not continuously. It was assumed that the acetaminophen and the metoclopramide were prescribed in the quantity of 10 tablets each for an outpatient malaria case, and that intravenous metoclopramide were prescribed over admission for an inpatient case, plus 10 tablets of acetaminophen and metoclopramide orally after discharge.

The cost of antimalarial drugs was provided by the Health Surveillance Foundation, and cost for analgesics and antiemetics drugs and for parenteral solutions for intravenous treatment by the Stock Prices of the Ministry of Health [28]. The personnel involved in malaria case management were a medical doctor for an outpatient case, plus nurse and nurse technician for an inpatient case. Reported time per consultation or per bed/day allocated to care for the each woman was 20 minutes for all professionals excluding nurse technicians (45 minutes). For inpatients, the cost spent on food, all meals (six meals/day) for the patient and for one companion was provided by a supplier of FMT-HVD.

Overhead costs data were retrieved from the Administration Department of the FMT-HVD based on reports of all economic and financial costs incurred during the year 2010 (S3 Table). Overheads included recurrent costs such as water, energy, cleaning, laundry, kitchen, communication, transportation, and other contracted services such as legal expenses, security, maintenance and capital costs. Capital costs included vehicles, at their replacement value, and capital investments such as the construction of new buildings or the restoration of old ones within the FMT-HVD facilities undertaken between 2005 and 2010. Useful life was considered as 30 years for buildings and 10 years for vehicles.

### Data analysis

All data from patients were collected through standardized questionnaires and analysed using Stata software (version 12, College Station, Texas, USA) and Microsoft Excel. The time horizon was one year and the year of analysis from the patient perspective was 2011 and from the provider perspective was 2010.

### Patient costs

From the patient perspective, direct costs (financial costs) were broken down into medical, transportation and other costs (food, phone calls, etc). Indirect costs were calculated by multiplying reported time lost by the nominal value of the median monthly permanent income per capita of households of Manaus from Census of 2010 (US$ 237.50) [29]. Patient costs incurred at the FMT-HVD or at JAPHP, as well as for any treatment sought earlier for the same episode, were calculated.

Due to distribution skewness, the median and the interquartile range (IQR) were used to report cost estimates, instead of mean and standard errors. Yet, mean values are also shown to provide a comprehensive description of cost distributions. Bootstrap simulation with 1000 replications was carried out to deal with the issue of distribution skewness [30, 31].

### Provider costs

From the health provider perspective, costs were estimated using a mixed approach of bottom-up and top-down costing [32]. Bottom-up approach was used for treatment costs. These were calculated by multiplying the quantity of each drug by the relative unit costs plus the time spent by each professional times the respective unit wage cost.

Top-down and bottom-up costing was also used to estimate the cost of a thick smear. The cost of a microscope, its maintenance and the training of a microscopist per slide examined was calculated by dividing the value of each input (considering that four microscopes and eight microscopists were the available resources for malaria diagnosis at the Malaria Diagnosis Department in the FMT-HVD) by the number of thick smears performed at the FMT-HVD in 2010 (27,257 thick smears) [11]. Personnel costs, estimated through bottom-up approach, were added to these figures.

Top-down costing was the approach used on overheads. On the 2010 FMT-HVD activity report, the number of basic care consultations (not involving a medical doctor), medical consultations, day hospital stays and hospital admissions were identified. To apportion overhead costs to one malaria episode as outpatient and as inpatient, all activities were expressed in terms of outpatient by assuming that a day of admission and a day hospital at the FMT-HVD correspond to 2, and a basic care consultation to half outpatient consultation, respectively. Total overheads were divided by the resulting number of outpatient-translated activities to estimate the share of overhead imputable to one outpatient consultation; this ratio was then multiplied by 2 and by 3 (average number of days for malaria inpatient) to impute overheads to an inpatient case. Overhead costs were distinguished between recurrent, such as annual general expenses, and capital costs, such as vehicles and long term investments.

Costs at the JAPHP were estimated based on one microscope, one microscopist, and one clinical technician for malaria diagnosis, with the clinical technician being also the focal person for administration of malaria treatment if the thick smear was positive.

Among non-overhead costs, personnel costs were considered as economic costs, while the remaining costs were considered as financial.

For sensitivity analysis parameters were varied 10% above and below these base-case values. For malaria diagnosis, upper limit of variation considered Macauley’s estimates of a passive plus active case detection diagnosis scenario; lower limit was the cost estimated by Malaria Laboratory of the Instituto Evandro Chagas (IEC) [19].

### Provider total costs in Manaus

Total provider cost for outpatient and inpatient MiP episodes reported at the FMT-HVD in 2010 were calculated by multiplying the unit costs of every resource used for diagnostic and treatment by the number of MiP cases. Total costs were calculated separately for *P*. *vivax* and *P*. *falciparum*. In 2010, 135 out of the total of 364 MiP episodes reported in the Manaus area presented at the FMT-HVD, with 120 of them treated as outpatients and 15 requiring admission. One hundred of the outpatient cases were due to *P*. *vivax* and 20 to *P*. *falciparum*. All the 15 malaria admissions were due to *P*. *vivax*. Total costs also included malaria screening of suspected cases that resulted negative (N = 298 at the FMT-HVD in pregnant women, year 2010). Total costs of treating MiP for the whole Manaus area, not only FMT-HVD, were calculated by considering that the number of malaria cases in pregnant women reported to other health facilities in the area was 229, of which 222 cases were due to *P*. *vivax* and 7 cases due to *P*. *falciparum*. Costs of the 229 MiP cases were estimated based on the assumption that these were managed at primary health facilities similar to the JAPHP: costs of JAPHP were, thus, used. All costs were expressed in US$ 2011. The average exchange rate Brazilian Real/US$ of 0.57 (January 2011 –March 2012) was applied [33]. Costs were adjusted from 2010 to 2011 based on inflation rate of 13%

## Results

A total of 73 malaria episodes in 64 women were included in the study between January 2011 and March 2012, with a refusal rate among those who were invited of 0%. Ten out of the 64 women enrolled were interviewed twice as they presented more than one positive thick blood smear during study period; in only one of them the time interval between interviews was less than seven days, considered as the same episode of the first interview, and not included in the analysis. In nine the mean interval was of 79.5 days (SD 42.63), then considered as new episodes and included in the analysis.

### Characteristics of study subjects

**Fig 1:**
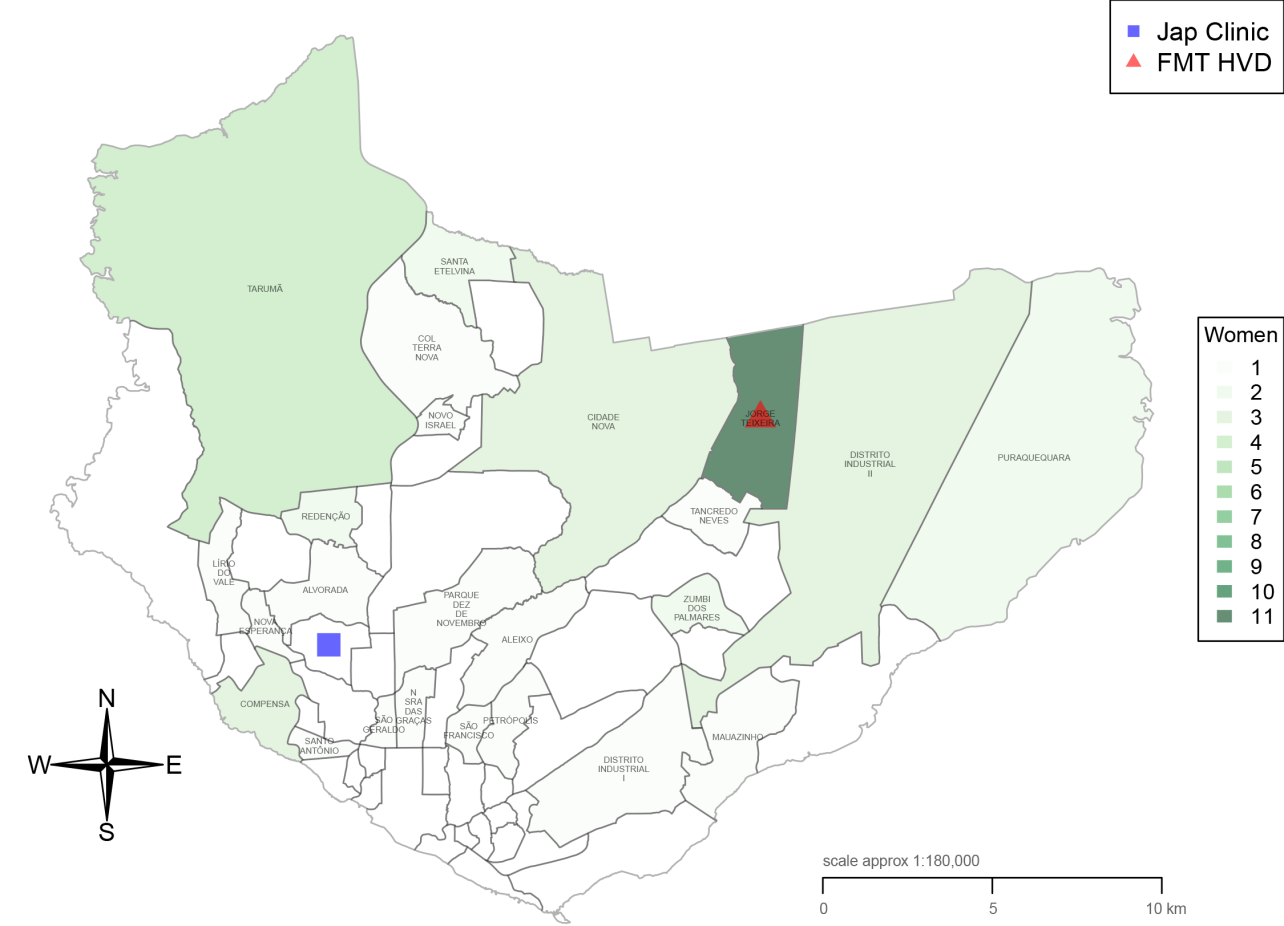
Manaus municipality and neighbourhoods of residence of women participating in the study.

**Fig 2:**
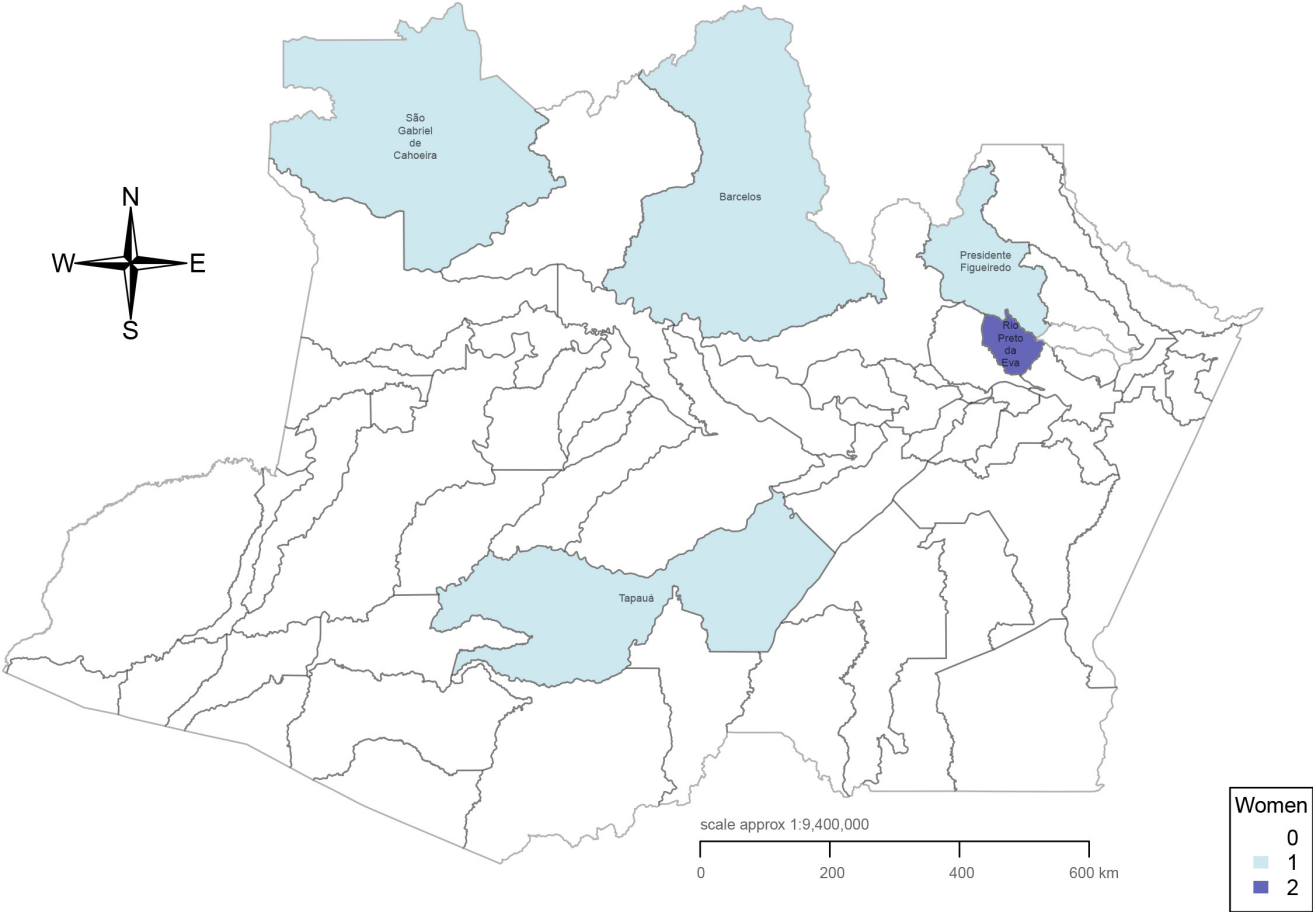
Amazonas state and region of residence of women participating in the study.

Table 1 shows the characteristics of study women. Nearly half of the women (47%; 30/64) were between 20 and 29 years of age, and a high proportion (20%; 13/64) were adolescents (≤ 19 years old), with the youngest aged 11 years. Most women (67%; 43/64) were from the Manaus urban area, and their main activity was housework (72%; 46/64). Nearly 50% of study women (30/64) came from two metropolitan areas of the city of Manaus where the incidence of malaria is reported to be highest, namely Jorge Teixeira and Tarumã areas [34] (Fig 1). Some of the women enrolled were from outside the Manaus area. Precisely, three of them were from nearby municipalities (Presidente Figueiredo and Rio Preto da Eva) and other three from very remote municipalities (São Gabriel da Cachoeira, Barcelos and Tapauá) (Fig 2). The median distance from place of residence to the health facility was 8.75 km, with the mean at 71.26 km and the maximum at 850 km. Maximum distance was travelled by an indigenous woman from a Yanomami tribe of São Gabriel da Cachoeira municipality.

**Table 1:** Baseline characteristics of study women, Manaus, Brazil, 2011–2012.

Characteristics (N = 64)		n	%
Age (years)	10 to 19	13	20
	20 to 29	30	47
	30 to 39	20	31
	≥ 40	1	2
Marital status	Common-law wife	35	55
	Married	12	19
	Single	17	27
Are of residence	Urban	43	67
	Rural	21	33
Main activity	Housewife	46	72
	Peasant	2	3
	Other formal occupation	15	23
	Student	1	2
Number of children^a^	0	25	40
	1 to 3	28	44
	4 to 7	11	16
		**Median**	**IQR**^b^
Number of malaria episodes per woman		1	1, 2
Time taken to reach the health facility (hours)^c^		1	0.67, 1.5
Estimated distance between home and the health facility (km)^d^		7.87	5.6, 24.5

^a^Excluding the current one.

^b^Interquartile range (lower and upper quantiles).

^c^Minimum time was 5 minutes and maximum 72 hours.

^d^Average distance was 71.26 km, minimum was 1.4 and maximum was 850.

### Characteristics of malaria episodes of study subjects

Table 2 summarizes malaria episodes characteristics. Malaria episodes included were homogenously distributed throughout the study period. Of the 73 episodes, 96% (70/73) were due to *P*. *vivax* and the remaining 4% (3/73) due to *P*. *falciparum*. Ninety two percent of episodes (67/73) was reported at the FMT-HVD, and 8% (6/73) at the JAPHF. The majority of episodes (89%; 65/73) were conducted as outpatient consultations. Six out of eight admissions (75%) were due to *P*. *vivax* infection.

**Table 2:** Characteristics of the malaria consultations and admissions, Manaus, Brazil, 2011–2012.

Characteristics (N = 73)		N	%
*Plasmodium* species	*P*. *vivax*	70	96
	*P*. *falciparum*	3	4
Women status at the time of interview	Pregnant	69	95
	Postpartum	4	5
Place of interview	FMT-HVD^a^	67	92
	Health facility^b^	6	8
Type of case in relation to *Plasmodium* species	Inpatient *P*. *vivax*	6	8
	Inpatient *P*. *falciparum*	2	3
	Outpatient *P*. *vivax*	64	88
	Outpatient *P*. *falciparum*	1	1
Malaria prevention tools used	Bed net	4	6
	Drugs^c^	9	12
	Skin repellent	14	19
	Insecticide	19	26
	No prevention	27	37
Transportation to the health facility	Bus	37	51
	Own car/motorbike	20	27
	Taxi	5	7
	Boat	2	0.03
	Other^d^	9	15
		**Median**	**IQR**^e^
Duration of clinical symptoms (days)^f^		5	3, 7
Number of nights admitted (if inpatient)^g^		3	2.5, 3.5

^a^Fundação de Medicina Tropical Doutor Heitor Vieira Dourado.

^b^João Avelino Pereira Health Facility.

^c^Prophylaxis with chloroquine.

^d^It includes walking and lift from someone.

^e^Interquartile range (lower and upper quantiles).

^f^Minimum duration was 2 days and maximum was 17.

^g^Minimum number was 1 night and maximum was 6.

The reported use of bed nets by study women was low (6%; 4/73) and 12% (9/73) reported having taken weekly chloroquine as a prevention of malaria relapse. The use of insecticides and skin repellents was reported in 26% (19/73) and 19% (14/73) of interviews, respectively. No use of malaria preventative measures was reported in nearly 40% of interviews (27/73). Bus was the transportation more frequently used (51%; 37/73) to reach the health facility, followed by car/motorbike (27%; 20/73), and taxi (7%; 5/73). Two study women travelled by boat along the Negro river. Six women enrolled at the FMT-HVD sought previous treatment at a pharmacy, while two women enrolled at the JAPHP reported having visited another health facility previously.

### Patient costs

Table 3 shows the costs per malaria outpatient consultation or admission, both including and excluding previous treatments eventually sought by the study women. Mean prevention costs were US$ 13.50 for an outpatient malaria consultation and US$ 3.85 for an admission. Out-of-pocket transportation median costs were US$ 10.66 (IQR, 5.33, 23.68) for a consultation and US$ 20.42 (IQR, 1.33, 51.8) for an admission. Unit cost of transportation, per km, was US$ 1.81 on average, with a median of US$ 0.66 (IQR, 0.61, 1.92). Total median costs including previous treatments sought were US$ 45.91 (IQR, 28.44, 80.41) and US$ 216.29 (IQR, 138.36, 327.07) for a consultation and an admission, respectively. The bootstrap analysis revealed that the median total costs including previous treatments sought were US$ 49.27 (IQR, 28.44, 80.41) for an outpatient consultation, and US$ 240.78 (IQR, 149.80, 393.42) for an admission. Table 4 shows costs incurred by the nine women who experienced two episodes of MiP during the study period. Median costs incurred at the health facility were US$ 259.50 (IQR, 120.69, 398.31) when the first malaria episode required admission and the second one was an outpatient consultation; median costs were US$ 25.38 (IQR, 22.79, 68.58) when the two malaria episodes were outpatient consultations. Interestingly, all women experiencing two episodes during the study period had *P*. *vivax* malaria. When previous treatments were sought median costs increased to US$ 335.85 (IQR, 273.38, 398.31) and to US$ 75.03 (IQR, 64.39, 90.33), respectively.

**Table 3:** Costs per malaria episode incurred by the pregnant women (US$ 2011).

Costs per malaria episode		Outpatient Consultations^a^ N = 65			Admissions^b^ N = 8		
		(*P*. *vivax* n = 64; *P*. *falciparum* n = 1)			(*P*. *vivax* n = 6; *P*. *falciparum* n = 2)		
		Mean	Median	IQR^c^	Mean	Median	IQR^c^
Deterministic analysis	Prevention costs	13.50	0	0, 11.54	3.85	0	0, 4.14
	Costs of previous symptomatic treatment sought	2.98	0	0, 2.66	15.34	0.81	0, 7.25
	Transportation costs to the health facility	17.64	10.66	5.33, 23.68	40.59	20.42	1.33, 51.8
	Other costs at the health facility^d^	1.76	0	0, 2.96	1.26	0	0, 1.48
	Medical costs for treatment at the health facility^e^	2.96	0	0, 1.78	0	0	0
	Indirect costs at the health facility^f^	3.04	2.94	2.20, 3.67	110.11	105.71	88.09, 123.33
	Indirect costs including previous treatments sought^g^^h^	49.11	19.94	3.43, 56.52	170.36	126.26	6105.71, 199.67
	Total costs including previous treatments^i^^h^	76.87	45.92	28.44, 80.41	242.38	216.29	138.36, 327.07
Bootstrap analysis^j^	Total costs including previous treatments^i^^h^	74.30	49.27	28.44, 80.41	245.13	240.78	149.80, 393.42

^a^Include cost until the moment of malaria diagnosis (first outpatient consultation).

^b^Include cost until the moment of the discharge.

^c^Interquartile range (lower and upper quantiles).

^d^These include cost for food, drink and phone calls.

^e^These include laboratory fees and drugs not provided at the health facility.

^f^These include the value of time lost at the health facility and money spent to contract a substitute in the main economic activity.

^g^These include the value of time lost during the whole pattern of treatment sought and money spent to contract a substitute in the main economic activity.

^h^Including previous symptomatic treatments sought.

^i^These include all costs, direct and indirect, associated with treatment incurred at the health facility plus direct and indirect costs eventually previously incurred for the same episode.

^j^Bootstrap based on 1000 replications.

**Table 4:** Costs for the treatment of *P*. *vivax* malaria incurred by women experiencing two episodes during study period (US $2011).

First episode	Second episode	Costs included	Number of women	Mean	Median	IQR^a^
Admission^b^	Outpatient consultation^c^	At the health facility	2	259.50	259.50	120.69, 398.31
Admission^b^	Outpatient consultation^c^	Including previous symptomatic treatments	2	335.85	335.85	273.38, 398.31
Outpatient consultation^c^	Outpatient consultation^c^	At the health facility	7	43.76	25.38	22.79, 68.58
Outpatient consultation^c^	Outpatient consultation^c^	Including previous symptomatic treatments	7	75.76	75.03	64.39, 90.33

^a^Interquartile range (lower and upper quantiles).

^b^Include cost until the moment of the discharge.

^c^Include cost until the moment of malaria diagnosis (first outpatient consultation).

### Provider costs

Tables 5 and 6 show unit and total costs for the diagnosis and treatment of MiP as outpatient and as inpatient at the FMT-HVD in 2010.

**Table 5:** Provider cost for outpatient malaria episodes in pregnant women reported in the FMT-HVD in 2010.

Provider cost for outpatient malaria episodes		Cost (US$ 2011)	*P*. *vivax* (n = 100)	*P*. *falciparum* (n = 20)	Total Cost^a^ (US$ 2011)
Malaria diagnose (N = 120)	Malaria test^b^^c^	6.44	644.00 (479.00, 760.00)	128.80 (95.80, 152.00)	772.80 (574.80, 912.00)
First outpatient consultation (N = 120)	Hemogram^b^	2.86	286.14	57.23	343.37
	Salaries of laboratory professionals^d^	11.59	1,159.00 (1,043.00, 1,275.00)	231.80 (208.60, 255.00)	1,390.80 (1,251.60, 1,530.00)
	Salaries of infectologist	14.91	1,491.00 (1,342.00, 1,641.00)	298.20 (268.40, 328.20)	1,789.20 (1,610.40, 1,969.20)
	Analgesics and antiemetics medicines^b^	0.285	28.50	5.70	34.20
	*P*. *vivax* treatment^b^^e^	0.171	17.10	-	17.10
	*P*. *falciparum* in the 1^st^ trimester treatment^b^^e^	4.2636	-	12.7908	12.7908
	*P*. *falciparum* after the 2^nd^ trimester treatment^b^^e^	1.8012	-	30.6204	30.6204
Second outpatient consultation (N = 120)^f^	Malaria test^b^^c^	6.44	644.00 (479.00, 760.00)	128.80 (95.80, 152.00)	772.80 (574.80, 912.00)
	Salaries of infectologist	14.91	1,491.00 (1,342.00, 1,641.00)	298.20 (268.40, 328.20)	1,789.20 (1,610.40, 1,969.20)
	Prophylaxis to prevent relapses^b^^g^	0.4104	41.04	-	41.04
Third outpatient consultation (N = 100)^h^	Malaria test^b^^c^	6.44	644.00 (479.00, 760.00)	-	644.00 (479.00, 760.00)
	Salaries of infectologist	14.91	1,491.00 (1,342.00, 1,641.00)	-	1,491.00 (1,342.00, 1,641.00)
	Hypnozoite form treatment^b^^i^	0.1596	15.96	-	15.96
Total			7,952.74 (6,894.74, 8,866.74)	1,192.14 (1,043.34, 1,321.74)	9,144.88 (7,938.08, 10,188.48)
Average direct cost per outpatient case^j^			79.53 (68.95, 88.67)	59.61 (52.17, 66.09)	76.21 (66.15, 84.90)
Indirect cost per outpatient case ^k^			23.98	23.98	23.98
Total cost per outpatient case			103.51 (92.93, 112.65)	83.59 (76.15, 90.07)	100.19 (90.13, 108.88)
Total cost per outpatient consultation^l^			34.50 (30.98, 37.55)	41.79 (38.07, 45.03)	

^a^Lower and upper level variation around the base-case.

^b^Financial costs.

^c^Considering the following inputs: thick smear test (glass slide, Giemsa and all the components for staining, immersion oil, lancet, cotton-wool, alcohol and gloves), microscope and maintenance, training for microscope technician, health agent and microscope technician time of work.

^d^Biochemist and laboratory technician

^e^Corresponds to treatment of 100 cases of *P*. *vivax*, 3 *P*. *falciparum* cases in the first trimester and 17 cases of *P*. *falciparum* after the second trimester.

^f^Consultation after seven days of the diagnose for check cure.

^g^In *P*. *vivax* cases only (N = 100)

^h^Consultation after 6 months of the delivery for only vivax cases

^i^Only primaquine if the result of the thick smear is negative.

^j^Considering malaria test, hemogram, salaries of FMT-HVD’s health professionals, analgesics and antiemetics medicines and malaria treatment.

^k^Considering overheads (recurrent costs such as water, energy, cleaning, contracted services such as security, maintenance and capital costs) divided by 159,705 consultations and procedures in FMT-HVD in 2010.

^l^Considering 3 consultations in *P*. *vivax* cases and 2 consultations in *P*. *falciparum* cases.

**Table 6:** Provider cost for inpatient malaria episodes in pregnant women reported in the FMT-HVD in 2010.

Provider cost for inpatient malaria episodes (N = 15)		Cost (US$ 2011)	Total Cost (US$ 2011)^a^
Malaria diagnose	Malaria test^b^^c^	6.44	96.60 (71.85, 114.00)
First day in Normal Ward	Hemogram^b^	2.86	42.92
	Salaries of laboratory professionals^d^	11.59	173.85 (156.45, 191.25)
	Salaries of infectologist	14.91	223.65 (201.30, 246.15)
	Salaries of nurses and nurse technicians	12.44	186.60 (167.85, 205.20)
	Nutrition^b^^e^	10.95	164.25
Second day in Normal Ward	Malaria test^b^^c^	6.44	96.60 (71.85, 114.00)
	Salaries of infectologist	14.91	223.65 (201.30, 246.15)
	Salaries of nurses and nurse technicians	12.44	186.60 (167.85, 205.20)
	Nutrition^b^^e^	10.95	164.25
Third day in Normal Ward	Malaria test^b^^c^	6.44	96.60 (71.85, 114.00)
	Salaries of infectologist	14.91	223.65 (201.30, 246.15)
	Salaries of nurses and nurse technicians	12.44	186.60 (167.85, 205.20)
	Nutrition^b^^e^	10.95	164.25
Treatment during hospitalization	Analgesics and antiemetics medicines^b^	3.5055	52.5825
	*P*. *vivax* in the 1^st^ trimester treatment^b^^f^	16.27179	32.54358
	*P*. *vivax* after the 2^nd^ trimester treatment^b^^f^	16.18059	210.34767
Outpatient consultation^g^	Malaria test^b^^c^	6.44	96.60 (71.85, 114.00)
	Salaries of infectologist	14.91	223.65 (201.30, 246.15)
	Prophylaxis to prevent relapses^b^^h^	0.4104	6.16
Final outpatient consultation^i^	Malaria test^b^^c^	6.44	96.60 (71.85, 114.00)
	Salaries of infectologist	14.91	223.65 (201.30, 246.15)
	Hypnozoite form treatment^b^^j^	0.1596	2.39
Direct cost per inpatient case^k^			211.64 (191.03, 229.82)
Indirect cost per inpatient case^l^			143.89
Total cost per inpatient case			355.53 (334.92, 373.71)
Total cost per inpatient case per day			118.51 (111.64, 124.57)
Total			3,174.59 (2,865.44, 3,447.29)

^a^Lower and upper level variation around the base-case.

^b^Financial costs.

^c^Considering the following inputs: thick smear test (glass slide, Giemsa and all the components for staining, immersion oil, lancet, cotton-wool, alcohol and gloves), microscope and maintenance, training for microscope technician, health agent and microscope technician time of work.

^d^Biochemist and laboratory technician.

^e^Patient and one companion.

^f^Corresponds to treatment of 15 cases of *P*. *vivax*, 13 cases after the second trimester and 2 cases in the first trimester. It was assumed that the intravenous treatment was necessary in three days of admission, ending the treatment with oral medicines.

^g^Consultation after seven days of the diagnose for check cure.

^h^In *P*. *vivax* cases only (N = 15).

^i^Consultation after 6 months of the delivery for only vivax cases.

^j^Only primaquine if the result of the thick smear is negative.

^k^Considering malaria test, hemogram, salaries of FMT-HVD’s health professionals, analgesics and antiemetics medicines, malaria treatment and nutrition.

^l^Considering overheads equals twice an outpatient visit and the lenght of hospitalization is three days.

The cost of one outpatient consultation for *P*. *vivax* malaria was US$ 34.50 (range US$ 30.98 to US$ 37.55) and US$ 41.79 (range US$ 38.07 to US$ 45.03) for *P*. *falciparum* malaria. The average cost of managing an outpatient case (multiple consultations) was US$ 100.19 (range US$ 90.13 to US$ 108.88), and discriminating by *Plasmodium* species, it was US$ 103.51 (range US$ 92.93 to US$ 112.65) for a *P*. *vivax* malaria episode and US$ 83.59 (range US$ 76.15 to US$ 90.07) for a *P*. *falciparum* malaria episode.

The cost of one bed/day was US$ 118.51 (ranging from US$ 111.64 to US$ 124.57) and considering the whole length of stay the cost of management of an inpatient case was US$ 355.53 (ranging from US$ 334.92 to US$ 373.71).

### Provider total costs in Manaus

Total provider costs for all malaria cases in pregnant women reported at the FMT-HVD in 2010 were US$ 12,479.73, of which US$ 11,287.60 (90.4%) were due to *P*. *vivax* malaria. Being the unit cost of a malaria diagnosis by microscopy of US$ 6.44, total cost of diagnosis for MiP in the same year, including both positive and negative cases, were US$ 2,788.52, of which US$ 1,919.12 associated with the negative cases and not included in the US$ 12,479.73 reported above.

Total provider costs for malaria cases in pregnant women reported to other health facilities in Manaus were US$ 4,558.77 in 2010, of which US$ 4,453.54 (97.7%) was due to *P*. *vivax* malaria.

Total provider costs for all malaria cases in pregnant women reported in Manaus in 2010 were US$ 17,038.50, of which US$ 15,741.14 (92.4%) was due to *P*. *vivax* malaria. Table 7 shows the provider total cost for malaria cases in pregnant women reported in Manaus in 2010 per health facility and per species.

**Table 7:** Provider total cost for all malaria cases in pregnant women reported in Manaus in 2010.

	Total		*P*. *vivax*			*P*. *falciparum*		
	N	Cost^a^	n	Cost^a^	%^b^	N	Cost^a^	%^b^
FMT-HVD^c^	135	12,479.73	115	11,287.60	90.4	20	1,192.13	9.6
Others HC^d^^e^	229	4,558.77	222	4,453.54	97.7	7	105.23	2.3
Total Manaus	364	17,038.50	337	15,741.14	92.4	27	1,297.36	7.6

^a^In US$ 2011.

^b^Of the costs.

^c^Fundação de Medicina Tropical Dr. Heitor Vieira Dourado.

^d^57 healths facilities other than FMT-HVD reported malaria cases in pregnancy in Manaus in 2010.

^e^These do not include indirect costs (overheads).

## Discussion

Malaria in pregnancy represents a relevant economic burden in this area of the Amazon region, despite being a low risk transmission setting where *P*. *vivax* predominates. The study reveals that the cost of treating malaria in pregnancy underwent a remarkable increase particularly when the patients required admission and in terms of indirect cost, and when the cost of subsequent *P*. *vivax* malaria episodes were considered. Patients’ costs reached up to 1.20% of the local average annual per capita income and the 3.4% when multiple episodes were taken into account. Considering that the income of pregnant women with malaria is likely to be lower than the average, these percentages may represent a lower bound of the actual economic burden to patients. From the health provider perspective, total costs for the management of all malaria cases in pregnancy in Manaus in 2010 approximately correspond to 30 times the average national government health per capita expenditure [35].

Radical treatment with primaquine, the unique hypnozoitocidal drug licensed, is contraindicated in pregnancy, making pregnant women more vulnerable to one or multiple relapses throughout gestation [36, 37]. In addition, any malaria episode exposes pregnant women to malaria paroxysm, which threats the outcome of pregnancy [2]. Thus, it could be argued that *P*. *vivax* malaria represents a dreadful extra toll by putting the mothers, and their offsprings, repeatedly at risk. In this study, it was not possible to differentiate if the subsequent malaria episodes suffered by women within the same pregnancy were a recrudescence, a relapse or a reinfection; though each of these additional episodes translates into additional economic costs to the health system and to the women.

According to study results the highest costs were incurred by women with *P*. *vivax* malaria, particularly when subsequent episodes were taken into account. In addition, being *P*. *vivax* the most prevalent species in the area, most of the costs for the treatment of MiP in the Brazilian Amazon are essentially associated with this species rather than with *P*. *falciparum*.

From the patient perspective, the costs data were obtained from a cross-sectional survey. Because of not using the longitudinal data method, it did not allow to estimating the whole cost of an episode of malaria arising from the complete path of care pregnant women receive, which consists of three consultations in the outpatient case or of an admission plus two consultations after discharge in the inpatient case. Therefore, patient costs estimated in the study represent only a share of the total economic burden women bore for one episode of MiP.

Study women reported low use and expenses on preventive tools. Insecticide-treated nets (ITNs) have recently been introduced as a malaria control tool in the area and distributed for free to the population. Therefore, at the time of the study, the few pregnant women reporting bed net use were very likely to be using a non-insecticide treated net instead of ITNs. Median cost on preventive tools among women who were admitted were lower than among those who were treated as outpatients, suggesting that lower expenditure in malaria prevention may be associated with a higher risk of severe disease.

Provider costs associated with the diagnosis and treatment of the 135 confirmed cases of malaria in pregnant women reported by the FMT-HVD in 2010 constituted 1% of the total activities undertaken that year in the tertiary hospital. However, costs associated with MiP are higher when costs of diagnostic tests performed to the rest of women with suspicion of malaria that resulted negative are considered. These costs (US$ 1,919.12) were higher than the total provider costs for the management of all *P*. *falciparum* malaria cases in pregnant women in 2010 in Manaus (US$ 1,297.37).

In Brazil, the recommended guidelines for MiP consist of active detection at each antenatal care visit and weekly prophylaxis with chloroquine to prevent relapses. In the current context of decreasing malaria incidence, both in the general population and in pregnant women [11, 38], questions are emerging on whether active case detection may remain cost-effective. Although this study did not include these costs as the focus was on the management of MiP confirmed cases, it offers invaluable information for cost-effectiveness analysis of the most adequate strategies for malaria in pregnancy control [39].

From a financial point of view, an important consideration is that funding from the National Unified Health System (SUS) was insufficient to ensure adequate resources for the management of malaria [40]. Funding to the FMT-HVD comes from the Amazonas state budget and from Brazil federal budget by the SUS. The SUS refunds fixed amounts according to procedure. For example, FMT-HVD receives for a malaria outpatient consultation performed by a medical doctor the value of US$ 5.7. This value is only the 5.69% of the total estimated cost. For an inpatient case, the FMT-HVD receives from the SUS the value of US$ 130.65, which in our estimates corresponds approximately to the cost of only one bed/day [US$ 118.51 (ranging from US$ 111.64 to US$ 124.57)], thus to 1/3 of the total cost of an average three days admission. In recent years, reductions in the federal share of SUS financing have been partly balanced by increased state and municipal health spending: in 2010, the Amazonas state assigned the 12% of the state budget to health, corresponding to approximately US$ 752.4 million [40].

The study mentioned specific aspects of healthcare services in the Amazon region, one of them being the Brazilian indigenous health system. Over the past years, the Brazilian government faced several difficulties in managing the indigenous healthcare services, and health care has been provided by different organizations [41]. Only in 2010 with the creation of the Indigenous Health Special Secretariat (SESAI), the Ministry of Health started to directly manage the healthcare of indigenous people [41, 42]. The indigenous woman that was enrolled in the study lived in a Yanomami village within the Pico da Neblina National Park, located in the municipality of São Gabriel da Cachoeira, 850 kilometers far from Manaus. She was transferred to Manaus by boat along the Negro river, a common situation in Amazonas state. A peculiarity in the access to specific health services in the Amazon region, not specific to indigenous population, is the need to travel long distances. During pregnancy, this can worsen maternal conditions, with possible negative consequences to the mother and fetus and increases costs for both patients and the health service.

A few studies previously engaged with the economics of malaria in Brazil. In the 90s, the average individual costs associated with malaria in Brazilian gold mining areas represented approximately 12% of the median annual salary [17]. In a mining town of the Brazilian Amazon, a programme consisting of all community members being offered free diagnosis with rapid tests and treatment with mefloquine, lead to conspicuous savings of 77% compared to passive case detection with quinine plus doxycycline, the recommended antimalarial drugs used as first line treatment [16, 18]. The overall cost-effectiveness of another malaria control programme including both vector control and case management over a period of seven years was US$ 69 per Disability-Adjusted Life Years averted. However, case treatment was found to be more cost-effective than vector control, particularly where *P*. *falciparum* was more prevalent than *P*. *vivax* and insecticide spraying was no more effective [13]. In another study, the aggregate productivity loss because of malaria for the years 1990–1994 in the city of Manaus was estimated to be about US$ 15.000 [14]. More recently, malaria diagnosis with light microscopy has been found to be more cost-effective than rapid diagnostic tests even in remote areas of the Amazonian basin if the accuracy of microscopists is maintained high [19]. None of these studies included data on pregnant women as a target population, nor their objectives are comparable to the ones of the present study.

In conclusion, this study reveals the high economic burden associated with *P*. *vivax* malaria in pregnancy in an area of low risk malaria transmission of Brazil. Especially when multiple infections are considered, costs associated with *P*. *vivax* are higher than costs associated with *P*. *falciparum*. The information generated will be of help for informed-based decision making for malaria in pregnancy control and elimination strategies in the region.

## Supporting Information

S1 Table Provider direct cost components of malaria diagnosis in pregnant women, Manaus, 2011–2012. (PDF)

S2 Table Provider direct cost components of malaria case management in pregnant women in FMT-HVD, Manaus, 2011–2012. (PDF)

S3 Table Provider overheads costs of the FMT-HVD based on reports of costs incurred during the year 2010. (PDF)

S1 Appendix Questionnaire administered to patients. (PDF)

## References

[1] Desai M., ter Kuile FO, Nosten F, McGready R, Asamoa K, Brabin B, et al, Epidemiology and burden of malaria in pregnancy. Lancet Infect Dis. 2007; 7: 93–104. 17251080 10.1016/S1473-3099(07)70021-X

[2] Chagas EDS, Nascimento CT, de Santana Filho FS, Bôtto-Menezes C, Martínez-Espinosa FE. Impact of malaria during pregnancy in the Amazon region. Rev Panam Salud Publica. 2009; 26: 203–208. 20058829 10.1590/s1020-49892009000900003

[3] Steketee RW, Nahlen BL, Parise ME, Menendez C. The burden of malaria in pregnancy in malaria-endemic areas. Am J Trop Med Hyg. 2001; 64: 28–35. 10.4269/ajtmh.2001.64.28 11425175

[4] Guyatt HL, Snow RW. Impact of malaria during pregnancy on low birth weight in sub-Saharan Africa. Clin Microbiol Rev. 2004; 17: 760–769. 15489346 10.1128/CMR.17.4.760-769.2004 PMC523568

[5] Bardají A, Sigauque B, Sanz S, Maixenchs M, Ordi J, Aponte JJ, et al Impact of malaria at the end of pregnancy on infant mortality and morbidity. J Infect Dis. 2011; 203: 691–699. 10.1093/infdis/jiq049 21199881 PMC3071276

[6] Ribera JM, Hausmann-Muela S, D'Alessandro U, Grietens KP. Malaria in pregnancy: what can the social sciences contribute? PLoS Med. 2007; 4: e92 17411326 10.1371/journal.pmed.0040092 PMC1851625

[7] Sachs J, Malaney P. The economic and social burden of malaria. Nature. 2002; 415: 680–685. 11832956 10.1038/415680a

[8] Worrall E, Morel C, Yeung S, Borghi J, Webster J, Hill J, et al The economics of malaria in pregnancy—a review of the evidence and research priorities. Lancet Infect Dis. 2007; 7: 156–168. 17251086 10.1016/S1473-3099(07)70027-0

[9] <Brasil. Guia prático de tratamento da malária no Brasil Brasília: Ministério da Saúde 2010.

[10] Oliveira-Ferreira J, Lacerda MV, Brasil P, Ladislau JL, Tauil PL, Daniel-Ribeiro CT. Malaria in Brazil: an overview. Malar J. 2010; 9: 115 10.1186/1475-2875-9-115 20433744 PMC2891813

[11] Pan American Health Organization. Report on the situation of malaria in the Americas. 2008.

[12] Brasil. Ministério da Saúde. Sistema de Informação de Vigilância Epidemiológica. Sivep-Malária. Available: http://portalweb04.saude.gov.br/sivep_malaria/default.asp. Accessed 2 January 2014.

[13] Akhavan D, Musgrove P, Abrantes A, d'A Gusmão R. Cost-effective malaria control in Brazil. Cost-effectiveness of a Malaria Control Program in the Amazon Basin of Brazil, 1988–1996. Soc Sci Med. 1999; 49: 1385–1399. 10509828 10.1016/s0277-9536(99)00214-2

[14] Alecrim JKC. Os impactos econômicos da malária na cidade de Manaus, MSc Thesis, 1995. Departamento de Economia e Análise, Universidade Federal do Amazonas, Manaus.

[15] Castilla RE, Sawyer DO. Malaria rates and fate: a socioeconomic study of malaria in Brazil. Soc Sci Med. 1993; 37: 1137–1145. 8235753 10.1016/0277-9536(93)90253-z

[16] Cunha ML, Piovesan-Alves F, Pang LW. Community-based program for malaria case management in the Brazilian Amazon. Am J Trop Med Hyg. 2001; 65: 872–876. 11791990 10.4269/ajtmh.2001.65.872

[17] Vosti SA. Malaria among gold miners in southern Para, Brazil: estimates of determinants and individual costs. Soc Sci Med. 1990; 30: 1097–1105. 2363060 10.1016/0277-9536(90)90296-5

[18] Pang LW, Piovesan-Alves F. Economic advantage of a community-based malaria management program in the Brazilian Amazon. Am J Trop Med Hyg. 2001; 65: 883–886. 11791992 10.4269/ajtmh.2001.65.883

[19] de Oliveira MR., de Castro Gomes A, Toscano CM. Cost effectiveness of OptiMal(R) rapid diagnostic test for malaria in remote areas of the Amazon Region, Brazil. Malar J. 2010; 9: 277 10.1186/1475-2875-9-277 20937094 PMC2959076

[20] de Oliveira MR., Giozza SP, Peixoto HM, Romero GA. Cost-effectiveness of diagnostic for malaria in Extra-Amazon Region, Brazil. Malar J. 2012; 11: 390 10.1186/1475-2875-11-390 23176717 PMC3533805

[21] Montekio VB, Medina G, Aquino R. Sistema de salud de Brasil. Salud Publica Mex. 2011; 53: s120–131. 21877078

[22] World Bank. Program Information Document (Appraisal Stage)—Service Delivery and Fiscal Management. Latin America and Caribbean. Available: http://www-wds.worldbank.org/external/default/WDSContentServer/WDSP/IB/2015/03/19/000442464_20150319092846/Rendered/PDF/951120PGID0P1532030Box385436B00PUBLIC0.pdf. Accessed 20 December 2015.

[23] Brasil. Indicadores e Dados Básicos (IDB). Available: http://tabnet.datasus.gov.br/cgi/idb2011/b09capc.htm. Accessed 20 December 2015.

[24] Brasil. Ministério da Saúde. Portal da Saúde Available: http://dw.saude.gov.br/portal/page/portal/sivep_malaria/. Accessed 28 July 2011.

[25] Amazonas. Diário Oficial do Estado do Amazonas 31.739. Poder executivo. Anexo II da Lei 3.469 de 24/12/2009. Available: http://www.imprensaoficial.am.gov.br/. Accessed 27 December 2013.

[26] Brasil. Instituto Brasileiro de Geografia e Estatística. Available: http://www.ibge.gov.br/home/. Accessed 29 December 2013.

[27] Macauley C. Aggressive active case detection: a malaria control strategy based on the Brazilian model. Soc Sci Med. 2005; 60: 563–573. 15550304 10.1016/j.socscimed.2004.05.025

[28] Brasil. Ministério da Saúde. Banco de Preços em Saúde. Available: http://aplicacao.saude.gov.br/bps/visao/consultaPublica/index.jsf. Accessed 29 December 2013.

[29] Brasil. Instituto Brasileiro de Geografia e Estatística. Censo Demográfico 2010. Available: http://www.censo2010.ibge.gov.br. Accessed 2 January 2014.

[30] Desgagné A, Castilloux AM, Angers JF, LeLorier J. The use of the bootstrap statistical method for the pharmacoeconomic cost analysis of skewed data. Pharmacoeconomics. 1998; 13: 487–497. 10180748 10.2165/00019053-199813050-00002

[31] Sicuri E, Davy C, Marinelli M, Oa O, Ome M, Siba P, et al The economic cost to households of childhood malaria in Papua New Guinea: a focus on intra-country variation. Health Policy Plan. 2012; 27: 339–347. 10.1093/heapol/czr046 21697246

[32] Mogyorosy Z, Smith P. The main methodological issues in costing health care services, in Centre for Health Economic Research Paper 7. 2005, University of York: York.

[33] OANDA. Available: http://www.oanda.com/currency/converter/. Accessed 1 September 2012.

[34] Alexandre MA, Ferreira CO, Siqueira AM, Magalhães BL, Mourão MP, Lacerda MV, et al Severe *Plasmodium vivax* Malaria, Brazilian Amazon. Emerg Infect Dis. 2010; 16: 1611–1614. 10.3201/eid1610.100685 20875292 PMC3294402

[35] World Health Organization. Available: http://apps.who.int/gho/athena/data/GHO/WHS7_156,WHS7_105,WHS7_104,WHS7_108.html?profile=ztable&filter=COUNTRY:*;REGION:*. Accessed 1 September 2012.

[36] Fernando D, Rodrigo C, Rajapakse S. Primaquine in vivax malaria: an update and review on management issues. Malar J. 2011; 10: 351 10.1186/1475-2875-10-351 22152065 PMC3306765

[37] Price RN, Douglas NM, Anstey NM, von Seidlein L. *Plasmodium vivax* treatments: what are we looking for? Curr Opin Infect Dis. 2011; 24: 578–585. 10.1097/QCO.0b013e32834c61e3 21986614 PMC3280893

[38] Pan American Health Organization. Report on the situation of malaria in the Americas. 2012.

[39] Sabot O, Cohen JM, Hsiang MS, Kahn JG, Basu S, Tang L, et al Costs and financial feasibility of malaria elimination. Lancet. 2010; 376: 1604–1615. 10.1016/S0140-6736(10)61355-4 21035839 PMC3044845

[40] Paim J, Travassos C, Almeida C, Bahia L, Macinko J. The Brazilian health system: history, advances, and challenges. Lancet. 2011; 377: 1778–1797 10.1016/S0140-6736(11)60054-8 21561655

[41] Brasil. Fundação Nacional de Saúde. Política Nacional de Atenção à Saúde dos Povos Indígenas. - 2ª edição—Brasília: Ministério da Saúde. Fundação Nacional de Saúde, 2002, Available: http://bvsms.saude.gov.br/bvs/publicacoes/politica_saude_indigena.pdf. Accessed 9 February 2014.

[42] Brasil. Presidência da República Federativa do Brasil. Portal Brasil. Decreto n° 7.336, de 19 de outubro de 2010, Available: http://www.planalto.gov.br/ccivil_03/_Ato2007-2010/2010/Decreto/D7336.htm. Accessed 9 February 2014.

