# Supplementary material for: Costs Associated with Malaria in Pregnancy in the Brazilian Amazon, a Low Endemic Area Where Plasmodium vivax Predominates
Source: PLoS Negl Trop Dis. 2016 Mar 31;10(3):e0004494. doi: 10.1371/journal.pntd.0004494 (PMC4816546; doi:10.1371/journal.pntd.0004494)
Supplement: S1 Table — (PDF) [file pntd.0004494.s001.pdf]

Supporting Information

S1 Table. Provider direct cost components of malaria diagnosis in pregnant women, Manaus, 2011-2012.

**Items**	**Unit cost (US$ 2011)[a]**	**Information sources**
Thick blood smear (one)[b]	1.43 (0.23, 2.19)	Base-case: Macaulay, 2005<br>Variation: Macauley, 2005 and de Oliveira et al., 2010, based on IEC/SVS/MS.[b]
Microscope - one unit - annual value	600.69 (405.62)	Base-case: Health Surveillance Foundation<br>Variation: de Oliveira et al., 2010, based on IEC/SVS/MS.[c]
Microscope maintenance - annual	59.85 (342.00)	Base-case and variation: Contracted service of FMT-HVD
Microscope technician - one annual training[d]	14.25 (11.40, 17.10)	Base-case and variation: Central Public Health Laboratory of the Amazonas
Health agent wage (monthly)	885.37	Department of Health of the State of Amazonas
Microscope technician wage (monthly)	885.37	Department of Health of the State of Amazonas

[a]Unit cost considered in the base-case analysis and variation.

[b]Corresponds to the individual cost of one test considering the following inputs: glass slide, Giemsa and other dyes (all the components for staining), immersion oil, lancet, cotton-wool, alcohol and gloves.

[c]Malaria Laboratory of the Instituto Evandro Chagas (IEC), of Health Surveillance Service/Ministry of Health.

[d]One annual 160-hour training for the thick smear microscopy reading.
