# Supplementary material for: Costs Associated with Malaria in Pregnancy in the Brazilian Amazon, a Low Endemic Area Where Plasmodium vivax Predominates
Source: PLoS Negl Trop Dis. 2016 Mar 31;10(3):e0004494. doi: 10.1371/journal.pntd.0004494 (PMC4816546; doi:10.1371/journal.pntd.0004494)
Supplement: S2 Table — (PDF) [file pntd.0004494.s002.pdf]

Supporting Information

S2 Table. Provider direct cost components of malaria case management in pregnant women in FMT-HVD, Manaus, 2011-2012.

**Items**	**Unit cost (US$ 2011)[a]**	**Information sources**
Hemogram	2.86	Table of Procedures (Ministry of Health) [b]
Laboratory technician wage (monthly)	885.37	Department of Health of the State of Amazonas
Biochemist wage (monthly)	2485.85	Department of Health of the State of Amazonas
Infectologist wage (monthly)	3579.38	Department of Health of the State of Amazonas
Nurse wage (monthly)	2485.85	Department of Health of the State of Amazonas
Nurse technician wage (monthly)	885.37	Department of Health of the State of Amazonas
Chloroquine - one tablet 150 mg	0.02	Health Surveillance Foundation (Amazonas)
Quinine sulfate - one tablet 500 mg	0.18	Health Surveillance Foundation (Amazonas)
Clindamycin - one capsule 300 mg	0.13	Health Surveillance Foundation (Amazonas)
Artemether + Lumefantrine - one pack 24 tablets	1.80	Health Surveillance Foundation (Amazonas)
Quinine dihydrochloride - one ampoule 600mg/2mL	0.21	Health Surveillance Foundation (Amazonas)
Clindamycin - one vial 600mg/4mL	0.52	Health Surveillance Foundation (Amazonas)
Artesunate - one vial 60mg/mL	1.64	Health Surveillance Foundation (Amazonas)
Artemether - one ampoule 80mg/mL	1.00	Health Surveillance Foundation (Amazonas)
Primaquine - one tablet 15 mg	0.01	Health Surveillance Foundation (Amazonas)
Dextrose 5% - one bottle 500mL	0.50	Stock prices on health (Ministry of Health)
Dextrose 5% - one bottle 100mL	0.48	Stock prices on health (Ministry of Health)
Acetaminofen - one tablet 500 mg	0.01	Stock prices on health (Ministry of Health)
Metoclopramide - one tablet 10mg	0.02	Stock prices on health (Ministry of Health)
Sodium Chloride 0.9% - one ampoule 10mL	0.05	Stock prices on health (Ministry of Health)
Metoclopramide - one ampoule 5mg/mL	0.02	Stock prices on health (Ministry of Health)
Patient nutrition (one day)	5.28	Contracted service of FMT-HVD
Companion nutrition (one day)	5.67	Contracted service of FMT-HVD

[a]Unit cost considered in the base-case analysis.

[b]Source: Brasil. Sistema de Gerenciamento da Tabela de Procedimentos, Medicamentos e OPM do SUS. Available: http://sigtap.datasus.gov.br/tabela-unificada/app/sec/procedimento/exibir/0202020380/12/2013. Accessed 23 December 2013.
