# Supplementary material for: Costs Associated with Malaria in Pregnancy in the Brazilian Amazon, a Low Endemic Area Where Plasmodium vivax Predominates
Source: PLoS Negl Trop Dis. 2016 Mar 31;10(3):e0004494. doi: 10.1371/journal.pntd.0004494 (PMC4816546; doi:10.1371/journal.pntd.0004494)
Supplement: S3 Table — (PDF) [file pntd.0004494.s003.pdf]

Supporting Information

S3 Table. Provider overhead costs of the FMT-HVD based on reports, incurred during the year 2010.

**Overhead costs**	**(US$ 2011)**[a]
Recurrent costs	
Cleaning/laundry/kitchen/transportation/security	1,537,327.93
Mainteinance	354,174.33
Administration	288,133.24
General Invoices (electricity, telephone, water, internet)	1,388,110.75
Capital costs	
General investments[b]	11,514.02
Hospital investments[b]	214,550.02
Administration investments[b]	4,728.05
Vehicles[c]	31,747.90
**TOTAL**	**3,830,286.24**
**Unit cost per outpatient visit**[d]	**23.98**
**Unit cost per inpatient episode (3 days admission)**[e]	**143.90**

[a]Costs were adjusted to 2011 figures based on an inflation rate of 13.4%

[b]Annualized based on 20 years useful life

[c]Annualized based on 8.5 years useful life

[d]Unit cost divided by 159,705 consultations and procedures in FMT-HVD in 2010.

[e]Considering an hospitalization for malaria in pregnancy equals twice an outpatient visit.
