# Supplementary material for: Costs Associated with Malaria in Pregnancy in the Brazilian Amazon, a Low Endemic Area Where Plasmodium vivax Predominates
Source: PLoS Negl Trop Dis. 2016 Mar 31;10(3):e0004494. doi: 10.1371/journal.pntd.0004494 (PMC4816546; doi:10.1371/journal.pntd.0004494)
Supplement: S1 Appendix — (PDF) [file pntd.0004494.s004.pdf]

Supporting Information

S1 Appendix. Questionnaire administered to patients.

Serial Number: ________	**Patient Cost Questionnaire**<br>**Cost of malaria during pregnancy**	Interviewer Initials:

Reminders to interviewer

This questionnaire is designed to find out about all the costs associated with a pregnant/post partum women (maximum one month after delivery) **who has been at** the hospital with **presumed or confirmed malaria or with an episode of anaemia**. It should be asked to the pregnant/post partum woman when leaving the health facility.

1. Date of interview (dd / mm / yyyy) |__|__|/|__|__|/|__|__|__|__|

2. At which health facility is the interview taking place? |__|

(**1**= name of health facility; **2**=name of health facility; **3**=name of health facility)

3. Place of interview |__|

(**1**=outpatient clinic; **2**=inpatient ward)

4. Current status |__|

(**1**=pregnant; **2**=puerperium)

5. Have you been diagnosed of malaria? |__|

6. Which parasite was detected? (this question may imply checking the health card)

(**1**=P vivax; **2**=P falciparum; **3**=mixed-infection) |__|

7. Secondary diagnosis

|__|__|__|__|__|__|__|__|__|__|__|__|__|__|__|__|__|__|__|__|__|__|__|-

|__|__|__|__|__|__|__|__|__|__|__|__|__|__|__|__|__|__|__|__|__|__|__|__|

DEMOGRAPHIC DATA

8. Date of birth (dd / mm / yyyy) |__|__|/|__|__|/|__|__|__|__|

9. Age |__|__|

10. Is this/was this your first child? |__|

(**1**=yes; **2**=no; **3**=don't know)

If not:

11. How many children do you have (not considering this) |__|

12. Where do you live? |__|__|__|__|__|__|__|__|__|__|__|__|__|__|__|__|__|

(name of village, quarter, etc)

13. Do you live in a rural or urban area? |__|

(**1**=rural; **2**=urban; **3**=don't know)

14. Marital status |__|

(**1**=married; **2**=”common-law wife” (live together but not official); **3**=single; **4**=divorced; **5**=widow)

PREVENTION USED DURING PREGNANCY

15. Did you sleep under a bed net last night? |__|
(**1**=yes; **2**=no; **3**=don't know)

If yes:

16. Did you buy it? |__|
(**1**=yes; **2**=no; **3**=don't know)

17. Did you get it for free from a health centre? |__|
(**1**=yes; **2**=no; **3**=don't know)

18. Did anyone (relatives, friends, etc) give it to you? |__|
(**1**=yes; **2**=no; **3**=don't know)

19. Other way of acquisition (please specify)___________________________________

In case you paid for the bed net:

20. How much did you pay? |__|__|__|__|__|

21. Did you take antimalaria drugs for prevention? |__|

(**1**=yes; **2**=no; **3**=don't know)

If yes

22. Did you have to pay for them? |__|
(**1**=yes; **2**=no; **3**=don't know)

If yes

23. How much did you spend for antimalarial drugs during pregnancy? |__|__|__|__|__|

24. Did you use any skin repellent during pregnancy? |__|
(**1**=yes; **2**=no; **3**=don't know)

If yes

25. How much did you spend for skin repellents during pregnancy? |__|__|__|__|__|

Did you use any insecticides for spraying your house during pregnancy? |__|
(**1**=yes; **2**=no; **3**=don't know)

If yes

26. How much did you spend for insecticides during pregnancy? |__|__|__|__|__|

ABOUT YOUR ILLNESS

27. How many days have you been ill with this episode of malaria?

(Between 0 and 24 hours = 1 day) |__|__|

28. Did you receive any treatment before you came/were admitted at this facility? |__|
(**1**=yes; **2**=no; **3**=don't know)

If yes

29. Did you seek for treatment at a traditional healer? |___|
(**1**=yes; **2**=no; **3**=don't know)

If yes

30. How much did you spend for treatment at the traditional healer? |___|___|___|___|___|

31. Did you seek treatment at a dispensary? |___|
(**1**=yes; **2**=no; **3**=don't know)

If yes

32. How much did you spend for fees? |___|___|___|___|___|

33. How much did you spend for transportation? |___|___|___|___|___|

34. How much did you spend for drugs? |___|___|___|___|___|

35. Did you buy drugs at a pharmacy?
(**1**=yes; **2**=no; **3**=don't know)

If yes

36. How much did you spend? |___|___|___|___|___|

TRAVEL TO THIS HEALTH FACILITY

37. Did you travel to the health facility by bus? |___|
(**1**=yes; **2**=no; **3**=don't know)

If yes

38. How much did you pay? |___|___|___|___|___|

39. Did you travel to the health facility by you own car? |___|
(**1**=yes; **2**=no; **3**=don't know)

If yes

40. How much did you pay? |___|___|___|___|___|

41. Did you travel to the health facility by taxi? |___|
(**1**=yes; **2**=no; **3**=don't know)

If yes

42. How much did you pay? |___|___|___|___|___|

43. Did you travel to the health facility by other means? |___|
(**1**=yes; **2**=no; **3**=don't know)

If yes

44. please specify the mean ____

45. How much did you pay? |___|___|___|___|___|

46. How long did it take you to get here from your home? Hours |___|___| Minutes |___|___|

47. Will you go home in same way? |___|
(**1**=yes; **2**=no; **3**=don't know)

If not

48. please specify means of travel home ---

49. and please specify the approximate cost |___|___|___|___|___|

COST IN THE HEALTH FACILITY IN THE CASE OF ADMISSION

50. How many nights have you been admitted? |__|__|

51. Did you pay a fee for the hospital admission? |__|
(**1**=yes; **2**=no; **3**=don't know)

If yes

52. How much did you pay? |__|__|__|__|__|

53. Have you paid for drugs during your stay? |__|

(**1**=yes; **2**=no; **3**=don't know)

If yes

54. How much did you pay for drugs? |__|__|__|__|__|

55. Do you have to take extra medicines to finish the treatment at home? |__|
(**1**=yes; **2**=no; **3**=don't know)

In the Table below identify and list any drugs that the patient is expected to buy when they leave to finish treating this case of malaria.

**Other medicine and treatments**	**Provided by hospital 1=Yes 2=No**	**If not, bought already 1=Yes 2=No**	**If buy, where did you buy?***	**Price**	**Units and Presentation 1= capsules; 2=syrup; 3=tablets; 4=injections)**	**Take for how many days?**
ACTs (Coartem…)						
Amodiaquine						
Paracetamol						
Fansidar						
Cotrimoxazol						
Aminofilina						
Fenoximetil						
Amoxiciline						
Ferrours salt						
Folic acid						
Quinine						
Other 1 (specify)						
Other 2 (specify)						

* (**1**=Pharmacy of the hospital **2**=Private pharmacy **3**=public pharmacy **4**=street trader **5**=kiosk **6**= Other)

OTHER COSTS ASSOCIATED WITH VISIT/ADMISSION

56. Did you pay for phone calls? |__|

(**1**=yes; **2**=no; **3**=don't know)

If yes

57. How much did you pay? |__|__|__|__|__|

58. Did you pay for food and drink?

(**1**=yes; **2**=no; **3**=don't know)

If yes

59. How much did you pay for food and drink? |__|__|__|__|__|

60. Other expenditure directly related to your illness and health facility visit (Please Specify)____ |__|__|__|__|__|

COST IN THE HEALTH FACILITY IN THE OUTPATIENTS CASE

61. How much time have you spent at this health facility? Hours |__|__|Minutes|__|__|

62. Did you pay a fee for the outpatient visit? |__|

(**1**=yes; **2**=no; **3**=don't know)

If yes

63. How much did you pay? |__|__|__|__|__|

64. Have you paid for drugs during the visit? |__|

(**1**=yes; **2**=no; **3**=don't know)

65. During this episode which medicines have you already bought before coming to this health facility?

In the table below specify what you can about the drugs that have already been bought. (We are interested in drugs given or prescribed before arriving at this health facility)

Feel free to check this info where possible with the woman's health passport.

**Name Drug**	**Bought already 1=Yes 2=No**	**Where did you buy these? ***	**Price**	**Units and Presentation 1= capsules; 2=syrup; 3=tablets; 4=injections)**	**Take for how many days?**
Combination therapy					
Amodiaquine					
Paracetamol					
Fansidar					
Cotrimoxazol					
Aminofilina					
Fenoximetil					
Amoxiciline					
Ferrours salt					
Folic acid					
Quinine					
Other 1 (specify)					

Other 2 (specify)					

*(**1**=Pharmacy of the hospital **2**=Private pharmacy **3**=public pharmacy **4**=street trader **5**=kiosk **6**= Other)

66. did you have to pay for laboratory tests? |__|

(**1**=yes; **2**=no; **3**=don't know)

If yes

67. How much did you pay? |__|__|__|__|__|

68. Did you have to pay for consultation fees? |__|

(**1**=yes; **2**=no; **3**=don't know)

If yes

69. How much did you pay for consultation fees? |__|__|__|__|__|

INDIRECT COSTS

70. Which activities did you stop due to your illness – and for how long?

Activity	No. hours per day	No. of Days
1. Livestock		
2. Agriculture		
4. Trade		
5. Household activities / Children care		
6. Business		
7. Short-term contract, other income-generating activities		
8. Other salary-earning job		
9. Studies		

71. Did you have to pay someone else to do your activities while you were ill?

(1=yes, 2=no, 3=don't know) |__|

If yes

72. How much did you have to pay this person? |__|__|__|__|__|

Please **thank** the respondent for their time.

Reassure them that all the information that they have shared with us in this questionnaire is **confidential**.
